# Effect of Aminophenyl and Aminothiahexyl α-d-Glycosides of the *Manno*-, *Gluco*-, and *Galacto*-Series on Type 1 Fimbriae-Mediated Adhesion of *Escherichia coli*

**DOI:** 10.3390/biology2031135

**Published:** 2013-09-03

**Authors:** Claudia Fessele, Thisbe K. Lindhorst

**Affiliations:** Otto Diels Institute of Organic Chemistry, Christiana Albertina University of Kiel, Otto-Hahn-Platz 3-4, 24098 Kiel, Germany; E-Mail: cfessele@oc.uni-kiel.de

**Keywords:** Type 1 fimbriae, bacterial adhesion, carbohydrate specificity, synthetic α-glycosides, glycoarrays, adhesion-inhibition assays, toxicity

## Abstract

Adhesion of bacteria to the glycosylated surface of their target cells is typically mediated by fimbrial lectins, exposed on the bacterial surface. Among the best-investigated and most important fimbriae are type 1 fimbriae, for which α-d-mannopyranoside-specificity has been described. This carbohydrate specificity is mediated by the type 1 fimbrial lectin FimH. In this account, we have employed four different set-ups to assay type 1 fimbriae-mediated bacterial adhesion, including tailor-made glycoarrays. The focus of our study was on testing FimH specificity with regard to the glycone part of a glycosidic ligand by testing a series of synthetic α-mannosides, as well as α-glucosides and α-galactosides. Unexpectedly, it was found that in solution all tested aminothiahexyl glycosides inhibit bacterial adhesion but that this effect is unspecific. Instead it is due to cytotoxicity of the respective glycosides at high mm concentrations.

## 1. Introduction

*Escherichia coli* is a widely distributed bacterial species that is responsible for many serious infections. Urogenital infections, for example, are caused by uropathogenic *E. coli* (UPEC). In order to infect their target cells, UPEC first have to accomplish adhesion to the glycosylated cell surface and establish colonization of the cell surface [1,2]. The bacterial adhesion process is facilitated by adhesive organelles, called fimbriae. The best-investigated fimbriae are type 1 fimbriae, which are hair-like, 1–2 μm long, and ~7 nm wide protein structures on the bacterial cell surface [3,4]. Type 1 fimbriae are widely expressed by *E. coli* and constitute important virulence factors of uropathogenic strains. They are used to mediate attachment to specific niches in the urinary tract [5]. Thus, type 1 fimbriae have a well-established role in urinary tract infections and in addition have been implicated in neonatal meningitis and Crohn’s disease [6,7].

It has been shown that glycoproteins carrying one or more *N*-linked high-mannose type oligosaccharides serve as receptor molecules for type 1 fimbriae. Early studies by Sharon and Ofek *et al.* have revealed the affinities of various oligosaccharides of different complexity [8,9,10,11,12]. From these studies it can be concluded that the presentation of α-d-mannosyl moieties, which varies in different oligosaccharides, is important for binding to type 1-fimbriated bacteria. This assumption is also supported by recent literature on carbohydrate binding of selectins [13]. Additionally, many studies with type 1 fimbriated bacteria were performed using multivalent mannosides as carbohydrate ligands, such as glycodendrimers or neoglycoproteins [14,15,16,17,18,19,20,21,22,23,24]. In these cases, statistic multivalency can lead to high avidity of the respective ligands. More recently, type 1 fimbriae-mediated bacterial adhesion has been studied and inhibited employing an armada of various synthetic mannosides with differing non-carbohydrate aglycone moieties to achieve effective antagonists of FimH [25,26,27,28,29,30]. This work has been extensively reviewed [31,32].

Apparently, carbohydrate binding of type 1 fimbriae is mediated by the lectin FimH, which is located at the fimbrial tips [33]. FimH is a two-domain lectin with its pilin domain FimH_P_ anchoring the lectin at the tip of the type 1 fimbrial shaft and its lectin domain, FimH_L_, harboring the carbohydrate-binding site. X-ray analysis of FimH has shown that [34,35,36,37] exactly one α-d-mannosyl residue can be accommodated within the carbohydrate-binding pocket (β-glycosides do not fit into the binding site). The aglycone moiety of a natural oligosaccharide exerts additional interactions at the periphery of the carbohydrate-binding site [35]. Likewise, non-natural aglycone portions can be used to increase the affinity of a synthetic α-d-mannoside according to computer docking and biological testing [31,38]. This approach has been promising in the context of an anti-adhesion therapy against urinary tract infections [39,40,41,42]. However, relatively recently, it has been found that FimH is a lectin that can function according to a catch bond mechanism [43]. Tensile forces, flow, or shear force, respectively, induce an allosteric switch, that also involves the carbohydrate-binding site, which is rearranged to a conformation, which binds α-d-mannosides more strongly [44].

**Figure 1:**
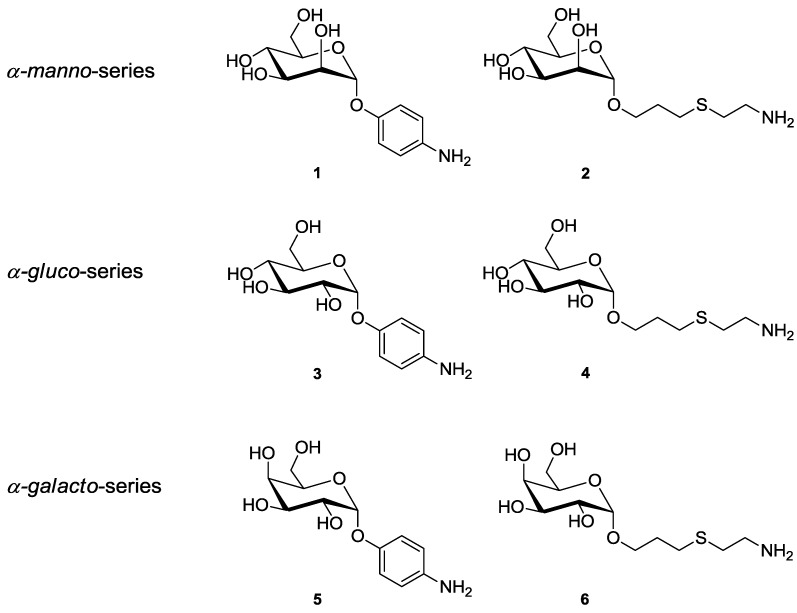
α-Glycosides of the *manno*-, *gluco*- and *galacto*-series, employed in the adhesion assays and for glycoarray fabrication. Nomenclature: 4-aminophenyl α-D-mannopyranoside (**1**, *p*APMan), 6-amino-4-thiahexyl α-D-mannopyranoside (**2**, ThiahexMan), 4-aminophenyl α-D-glucopyranoside (**3**, *p*APGlc), 6-amino-4-thiahexyl α-D-glucopyranoside (**4**, ThiahexGlc), 4-aminophenyl α-D-galactopyranoside (**5**, *p*APGal), 6-amino-4-thiahexyl α-D-galactopyranoside (**6**, ThiahexGal).

Thus, FimH can be considered as an especially intriguing lectin, with the potential to structurally rearrange its carbohydrate-binding site. This has prompted us to revisit inhibition of type 1-fimbriated bacterial adhesion employing a collection of six synthetic α-glycopyranosides of the *manno*-, *gluco*-, and *galacto*-series, **1**–**6** (Figure 1). Many other mannose-specific lectins are known to display a carbohydrate specificity for monosaccharides other than mannose [45]. However, in the case of FimH and type 1-fimbriated bacteria, respectively, glycosides other than mannosides were typically used as negative controls.

**Figure 2:**
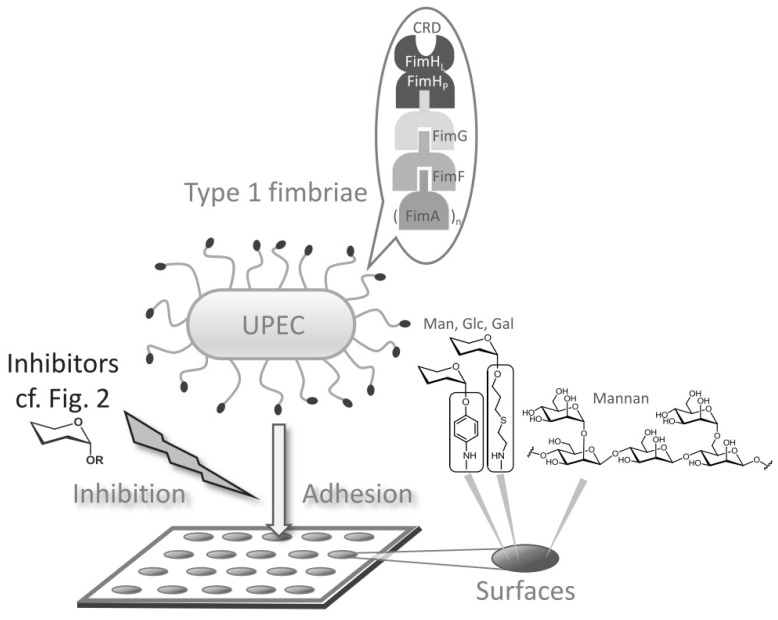
Type 1 fimbriae-mediated adhesion of uropathogenic *Escherichia coli* (UPEC) to a glycosylated surface is mediated by type 1 fimbriae. Type 1 fimbriae are rod-like adhesive organelles exposed by the bacteria, terminated by the lectin FimH for which a clear specificity for α-d-mannosides has been described. Here, it was tested if also glucosides and galactosides can exert some inhibitory potency in this system. As glycosylated surfaces mannan-coated polystyrene microtiter plates were used on the one hand, and alternatively, tailor-made microtiter plate-based glycoarrays were employed, comprising α-mannoside, α-glucoside, and α-galactoside functionalization. A corresponding series of glycosides were tested as inhibitors of bacterial adhesion in solution.

Carbohydrate specificity of lectins is typically tested in solution or on polystyrene microtiter plates, which can be carbohydrate-decorated to allow type 1 fimbriae-mediated adhesion of *E. coli* (Figure 2). The potencies of inhibitors of this adhesion process are mostly obtained from inhibition curves and expressed in the form of IC_50_ values. In our study, four different assays were employed: (i) A binding assay with GFP-tagged *E. coli* to microtiter plate-based glycoarrays testing varied bacterial concentration and (ii) varied glycoarray density; (iii) an adhesion-inhibition assay to test the prepared series of synthetic α-glycosides as inhibitors of bacterial adhesion to a mannan-coated surface in solution, and (iv) a preincubation-inhibition-adhesion assay in which the bacteria are allowed to interact with the glycosides in solution before they are transferred to the microplates. This approach should allow us to test if in any set-up an altered carbohydrate specificity of type 1 fimbriae-mediated bacterial adhesion can be seen.

## 2. Experimental Section

### 2.1. Glycoside Synthesis

All glycosides used for the bacterial adhesion assays (Figure 2, **1**–**6**) were synthesized according to known procedures. The aminophenyl glycosides, **1**, **3**, and **5**, were obtained by catalytic reduction from the corresponding nitrophenyl glycosides, which are commercially available. The 6-amino-4-thiahexyl glycosides, **2**, **4**, and **6**, where synthesized starting with the respective allyl glycosides by radical addition of cysteamine [46]. All prepared compounds were pure according to NMR and mass spectrometric analysis, and the analytical data obtained were in accordance with the literature.

### 2.2. Media and Buffer Solutions

Carbonate buffer solution (pH 9.2): sodium carbonate (1.59 g) and sodium hydrogen carbonate (2.52 g) were dissolved in distilled deionized water. PBS buffer solution (pH 7.2): sodium chloride (8.00 g), potassium chloride (200 mg), sodium hydrogen phosphate dihydrate (1.44 g), and potassium dihydrogen phosphate (200 mg) were dissolved in distilled deionized water (1.00 L). PBST buffer solution: PBS buffer + Tween^®^20 (0.05% v/v). Lysogeny broth (LB) medium: tryptone (10.0 g), sodium chloride (10.0 g), and yeast extract (5.00 g) were dissolved in distilled deionized water (1.00 L) and autoclaved, and ampicillin (100 mg) and chloramphenicol (50 mg) were added. Agar plates: tryptone (10.0 g), sodium chloride (10.0 g), yeast extract (5.00 g), and agar (15.0 g) were dissolved in distilled deionized water (1.00 L) and autoclaved, and ampicillin (100 mg) and chloramphenicol (50 mg) were added. All pH values were adjusted with HCl (1 m) and NaOH (1 m).

### 2.3. Bacterial Strain, Growth Conditions

For testing the GFP-expressing *E. coli* strain pPKL1162 was used [47]. This *E. coli* mutant exclusively expresses type 1 fimbriae as well as GFP (green fluorescent protein) allowing for quantification of bacterial adhesion by fluorescence read-out [46]. The *E. coli* strain pPKL1162 was constructed by inserting the pPKL1174 plasmid into strain SAR18. The pPKL1174 plasmid contains the *fim* gene cluster, which is responsible for the expression of type 1 fimbriae. SAR18 includes the GFP gene on its genome, controlled by a constitutive promoter [47]. The pPKL1162 strain was cultured from a frozen stock in LB medium and incubated overnight at 37 °C. After washing twice with PBS buffer (2.00 mL) the bacteria pellet was resuspended in PBS buffer and the bacterial solution was adjusted to OD_600_ = 0.4 (≙ 2 mg/mL) with PBS.

### 2.4. Mannan Coating of Microtiter Plates, Covalent Functionalization

Black 96-well microtiter plates (Nunc, MaxiSorp) were incubated with mannan from *Saccharomyces cerevisiae* (1.2 mg/mL in carbonate buffer, 120 µL/well) and dried overnight at 37 °C. After washing with PBST (3 × 150 µL/well) the wells were blocked with BSA (5% in PBS, 120 µL/well) and incubated at 37 °C for 2 h. Following this, the microtiter plates were washed with PBST (2 × 150 µL/well) and PBS (150 µL/well) [46].

### 2.5. Covalent Functionalization of Microtiter Plates: Glycoarray Preparation

For glycoarray preparation, black Immobilizer Amino™ F96 MicroWell™ plates (Nunc) were incubated with solutions of the respective glycosides in carbonate buffer (100 µL/well; concentrations of used glycoside solutions are indicated individually, see binding/inhibition curves) at ambient temperature and at 100 rpm overnight. Following washing with PBST (3 × 150 µL/well), the wells were blocked with ethanolamine (10 mm in carbonate buffer, 120 µL/well) and incubated at ambient temperature and 100 rpm for 2 h. Following this, the microtiter plates were washed with PBST (2 × 150 µL/well) and PBS (150 µL/well) [46].

### 2.6. Binding Assay with GFP-Tagged E. coli

(**a**) Variation of bacteria concentration: Glycoarrays were prepared with 10 mm of the respective glycoside in carbonate buffer and to these glycoside-functionalized microtiter plates PBS buffer was added (100 µL/well). Then, the prepared bacterial solution (OD_600_ 0.4) was added to well 1 and 2 and serially diluted (starting with well 2). The plate was incubated for 1 h at 37 °C, at 100 rpm, washed with PBS (3 × 150 µL/well), and then PBS was added (100 µL/well) and the fluorescence intensity (485 nm/535 nm) determined [46].

(**b**) Variation of glycoside concentration: Glycoarrays were prepared using serial dilutions of the respective glycoside (starting with 200 mm glycoside solution in carbonate buffer). Following this, the prepared bacterial solution (OD_600_ 0.4, 100 µL per well) was added and the plate incubated for 1 h at 37 °C at 100 rpm. The plate was washed with PBS (3 × 150 µL/well), and then PBS was added (100 µL/well) and the fluorescence intensity (485 nm/535 nm) determined [46].

### 2.7. Adhesion-Inhibition Assay with GFP-Tagged E. coli

Solutions of the respective inhibitory glycosides were prepared (200 mm carbonate buffer except for *p*APMan: 10 mм) and serial dilutions of the inhibitor solution added to the mannan-coated microtiter plate wells. The prepared bacterial solution (OD_600_ 0.4, 50 µL/well) was added and the plate incubated for 1 h at 37 °C and 100 rpm. The plates were washed with PBS buffer (3 × 150 µL/well), and then the wells were filled with PBS (100 µL/well) and the fluorescence intensity (485 nm/535 nm) was determined [46].

### 2.8. Preincubation-Inhibition-Adhesion Assay with GFP-Tagged E. coli

Serial dilutions of the respective inhibitory glycoside solution were prepared (starting concentration 200 mм in carbonate buffer except for *p*APMan: 10 mм) and mixed with the prepared bacterial solution (OD_600_ 0.4, 60 µL). /well) for preincubation. These mixtures were incubated for 1 h at 37 °C and 100 rpm and were then transferred to mannan-coated microtiter plates followed by incubation for 1 h at 37 °C and 100 rpm. The plates were washed with PBS buffer (3 × 150 µL/well), the wells filled with PBS (100 µL/well) and the fluorescence intensity (485 nm/535 nm) was determined [27].

### 2.9. Bacterial Growth Tests

Serial dilutions of the respective inhibitory glycoside solutions were prepared (starting concentration 200 mm in carbonate buffer) and mixed with a bacterial solution (OD_600_ 0.4, 50 µL /well). The resulting mixtures were incubated for 1 h at 37 °C and 100 rpm. Following this, 50 µL of each mixture were transferred to an agar plate, which were incubated overnight at 37 °C. Bacterial growth was determined by visual inspection of the agar plates.

## 3. Results

### 3.1. Binding of GFP-Tagged E. coli to Glycoarrays on Polystyrene Microtiter Plates

Glycoarrays have become valuable tools in glycobiology [48,49,50]. We, and others, have prepared glycoarrays on polystyrene microtiter plates to facilitate the study of cellular adhesion, including bacterial adhesion assays [51,52,53,54]. One of the classic approaches to study bacterial adhesion to carbohydrate surfaces is to employ mannan-coated microtiter plates. Mannan from *Saccharomyces cerevisiae* is a polydisperse polysaccharide that sticks to polystyrene surfaces by hydrophobic forces. It exposes α-d-mannosyl residues, which allow for attachment of type 1-fimbriated *E. coli* cells (Figure 2). However, when microtiter plates are functionalized using specific glycosides, the resulting glycoarray surfaces are more precisely defined than when mannan coating is employed. In addition, when synthetic glycosides are used for glycoarray fabrication, tailor-made surfaces can be designed to address specific questions of carbohydrate recognition and to test binding properties of lectins in great detail.

**Figure 3:**
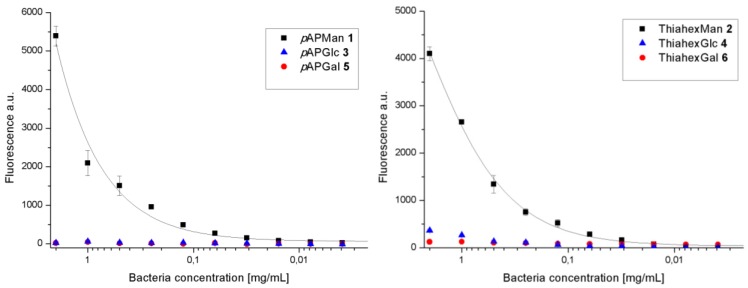
Bacterial binding to glycoarrays on microtiter plates with different bacteria concentrations. The depicted binding curves are representative examples from several (>3x) independent experiments. Error bars result from duplicate values on one plate.

The series of *manno*-, *gluco*- and *galacto*-configured α-d-glycosides we used carry aglycone portions modified with a terminal amino group (Figure 1). This allows us to chemically attach the respective glycoside to the surface of pre-activated polystyrene microtiter plates. Thus, glycosides **1**–**6** were individually immobilized onto the surface of microtiter plates using 10 mm concentrations in all cases. Following this, the fluorescent type 1-fimbriated *E. coli* bacteria were added in serial dilution to the resulting glycoarray-decorated plates. After washing of the plates bacterial adhesion was measured by readout of fluorescence intensity (Figure 3).

Glycoarrays prepared from the phenyl mannoside **1** or the alkyl mannoside **2** led to effective adhesion of bacterial cells in a perfect concentration-dependent manner, as expected. However, the glycoarrays prepared from glycosides of the *gluco*- and *galacto*-series, respectively, were not suited to mediate type 1 fimbriae-mediated bacterial adhesion. Indeed, no bacterial adhesion was detected. As we were interested to assess, if the carbohydrate specificity of type 1 fimbriae-mediated bacterial adhesion is strictly limited to α-d-mannosides, we tested glycoarrays at higher sugar concentrations in the next step.

**Figure 4:**
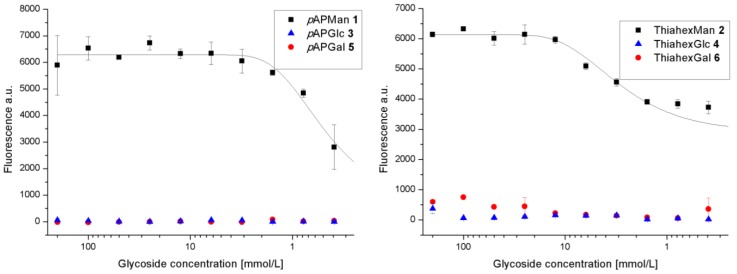
Bacterial binding to glycoarrays on microtiter plates. Various concentrations of glycosides were used for surface functionalization. The depicted binding curves are representative examples from several (>3x) independent experiments. Error bars result from duplicate values on one plate.

Thus, glycoside solutions used for glycoarray fabrication were serially diluted starting with a comparatively high concentration (200 mm). On the other hand, a constant number of fluorescent *E. coli* cells were used in the adhesion experiments. Fluorescence read-out showed a plateau of maximal adhesion efficiency at “densely” functionalized plate surfaces and diminishing bacterial adhesion starting at lower glycoside concentrations when mannosides **1** or **2** were used for the functionalization of the microtiter plates (Figure 4). Again, glycoarrays prepared from glucosides (**3** or **4**) or galactosides (**5** or **6**), respectively, did not lead to an adhesive surface.

### 3.2. Adhesion-Inhibition Assay with GFP-Tagged E. coli

In the next step of our study, all glycosides, which had been used before for glycoarray preparation (**1**–**6**), were now used as soluble inhibitors of type 1 fimbriae-mediated bacterial adhesion in solution. It was important to test, if the effect of the putative FimH ligands in solution was different from their effect as immobilized ligands. Hence, serial dilutions of inhibitor solutions were used to interfere with bacterial adhesion to mannan-coated microtiter plates. On each individual plate, we tested for methyl α-d-mannopyranoside (MeMan) to provide a universal reference inhibitor of type 1 fimbriae-mediated bacterial adhesion. Inhibition curves were determined for each tested inhibitor and IC_50_ values were deduced where possible. The IC_50_ value represents the inhibitor concentration at which 50% of bacterial adhesion is prevented. As MeMan was always simultaneously tested, relative inhibitory potencies (RIP values) can be specified for all tested glycosides with respect to the IC_50_ obtained for MeMan, which was defined as reference inhibitor potency with IP≡1.

**Figure 5:**
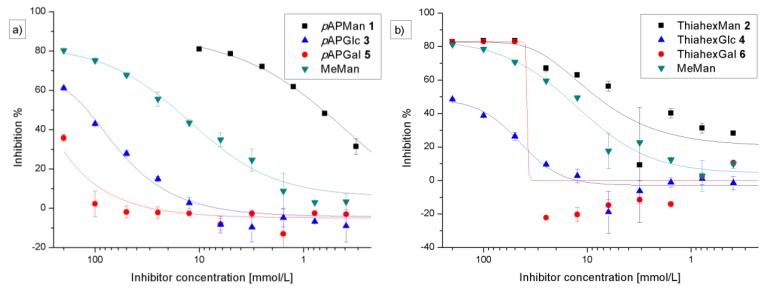
Inhibition curves obtained with the phenyl glycosides **1**, **3**, and **5** (**a**) and the alkyl glycosides **2**, **4**, and **6** (**b**) as inhibitors for type 1 fimbriae-mediated bacterial adhesion to mannan. The depicted inhibition curves are representative examples from several (>3x) independent experiments. The standard inhibitor MeMan was tested simultaneously on each plate. Error bars result from duplicate values on one plate.

As expected, the two mannosides **1** and **2** gave the best IC_50_ values in this assay. These results are in accordance with the literature [31]. Unexpectedly however, also the phenyl glucoside **3** showed an inhibitory potency, although it was smaller than that of MeMan (Figure 5a). Interestingly, the alkyl galactoside **6** showed an unexpected inhibitory effect when employed at concentrations greater than 25 mm. In the same concentration range, the alkyl glucoside **4** had a less pronounced effect (Figure 5b). Strikingly, at concentrations <25 mm galactoside **6** had no effect on bacterial adhesion at all. The obtained “curve” cannot be considered a regular inhibition curve.

### 3.3. Preincubation-Inhibition-Adhesion Assay with GFP-Tagged E. coli

In the adhesion-inhibition assay (*vide supra*) a soluble inhibitor has to compete against a highly mannosylated (mannan-coated) surface for binding to the type 1 fimbrial lectin. In contrast to this, in the preincubation-inhibition-adhesion assay [27], the uropathogenic *E. coli* were first incubated with the respective inhibitor under physiological conditions for 1 h. Only then, this suspension was transferred to the mannan-coated microtiter plate surface. In the adhesion-inhibition assay both ligands, mannan and the tested inhibitor, are presented to the bacteria at the same time. In contrast, preincubation of the bacteria with inhibitor solution can lead to saturation of the lectin’s carbohydrate binding sites prior to the adhesion experiment on the mannan-coated surface. Thus, in this assay bacterial binding to mannan is only possible if the fimbrial binding site is not effectively saturated with the inhibitor after preincubation.

**Figure 6:**
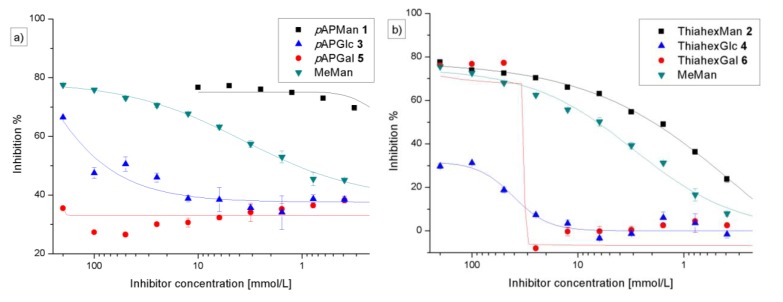
Preincubation-inhibition-adhesion assay with GFP-tagged *E. coli*: Inhibition of bacterial adhesion to mannan as obtained with the phenyl glycosides **1**, **3**, and **5** (**a**) and the alkyl glycosides **2**, **4**, and **6** (**b**) after preincubation with the bacteria. The depicted inhibition curves are representative examples from several (>3x) independent experiments. The standard inhibitor, MeMan, was tested on the same plate. Error bars result from duplicate values on one plate.

However, bacterial adhesion after preincubation with the inhibitory glycosides led to very similar results as the adhesion-inhibition assay without preincubation (Figure 6, Table 1). The galactoside **6** showed a similar effect as seen before (Figure 5b).

For a more detailed comparison, the testing results obtained in the adhesion-inhibition experiments without and with preincubation of bacteria are summarized in Table 1. Significant differences between the two assays can be seen mainly in the IC_50_ values for the mannosides **1** and **2**, as well as for the glucoside **3**. The glucoside **4** and the galactoside **5** could not effect 50% inhibition of bacterial adhesion in both tests and, therefore, no IC_50_ values could be determined. Owing to the unusual curves obtained with galactoside **6**, no IC_50_ values were deduced in this case.

**Table 1:** Inhibition of type 1 fimbriae-mediated *E. coli* adhesion to mannan-coated microtiter plates. For IC_50_ and RIP values are averaged from mean values from three independent tests.

	*Adhesion-inhibition assay*			*Preincubation-inhibition-adhesion assay*		
Glycoside	IC_50_ [mm] (SD)	RIP#(SD)	total inhibition %	IC_50_ [mm] (SD)	RIP#(SD)	total inhibition %
**1** (*p*APMan)	0.62 (0.21)	27.8 (1.65)	83	0.035 (0.02)	30.2 (7.8)	76
**2** (ThiahexMan)	5.28 (2.47)	2.41 (0.37)	80	1.19 (0.79)	3.46 (0.12)	78
**3** (*p*APGlc)	122 (1.99)	0.135 (0.01)	60	51.35 (27)	0.023 (0.01)	63
**4** (ThiahexGlc)	−	−	45	−	−	46
**5** (*p*APGal)	−	−	32	−	−	49
**6** (ThiahexGal)	−	−	77	−	−	75

# RIP values are relative inhibitory potencies based on the IC_50_ value measured for MeMan on the same plate.

### 3.4. Bacterial Growth Tests

As a next step, it was important to analyze the effect of galactoside **6** on bacterial adhesion, and to assess whether it is due to specific inhibition of fimbriae-mediated binding or is caused by other processes. Thus, highly concentrated solutions of **6** were incubated with the bacteria and then bacterial growth was tested on agar plates. Visual inspection of the agar plates after one day clearly showed that solutions of galactoside **6** at concentrations >25 mm were cytotoxic for the bacteria. No bacterial growth was observed in repeated experiments. After this finding, all other glycosides we used were tested in the same assay and the results are summarized in Table 2. According to this test, none of the tested *p*-aminophenyl glycosides (**1**, **3**, **5**) are cytotoxic at high concentrations. However, all aminothiahexyl glycosides (**2**, **4**, **6**) were cytotoxic at high concentrations, the glucoside **4** at concentrations ≥100 mm, galactoside **6** at concentrations ≥50 mm, and, surprisingly, mannoside **2** inhibits bacterial growth even at a concentration of 25 mm.

**Table 2:** Bacterial growth was tested on agar plates after1 h of incubation with glycosides **1**–**6**, which were employed at the indicated concentrations. + Bacteria grow; − no bacterial growth could be seen by visual inspection; n.t. not tested.

	*p*APMan **1**	*p*APGlc **3**	*p*APGal **5**	ThiahexMan **2**	ThiahexGlc **4**	ThiahexGal **6**
200 mM	+	+	+	−	−	−
100 mM	+	+	+	−	−	−
50 mM	+	+	+	−	+	−
25 mM	+	+	+	−	+	+
12.5 mM	n.t.	n.t.	n.t.	+	n.t.	n.t.

## 4. Discussion and Conclusion

To test the carbohydrate specificity of the bacterial lectin FimH under different conditions, pairs of α-configured synthetic glycosides of the *manno*-, but also the *gluco*- and *galacto*-series (**1**–**6**) were investigated with type 1 fimbriae-mediated bacteria. Two different aglycone moieties were used, namely an aromatic *p*-aminophenyl unit and the 6-aminothiahexyl spacer (Figure 1). When these glycosides were used for glycoarray fabrication on polystyrene microtiter plates, a strict mannose specificity was seen in type 1 fimbriae-mediated bacterial adhesion (Figure 3, Figure 4). The glucose-, as well as galactoside-decorated surfaces had no adhesive property in this adhesion system. However, when glucosides (**3** and **4**) and galactosides (**5** and **6**) were tested in solution as inhibitors of bacterial adhesion to a mannan surface, rather unexpectedly, an inhibitory effect was observed (Figure 5, Figure 6). The α-glucosides **3** and **4** showed a concentration-dependent inhibitory potency in both tests, in the adhesion-inhibition assay as well as in the preincubation-inhibition-adhesion assay. However, in both cases the determined inhibitory potency was weaker than that of MeMan and of the mannosides **1** and **2**. The phenyl galactoside **5** had no inhibitory potency, but the inhibitory effect of the aminothiahexyl galactoside **6** was comparable to that of the respective mannoside **2** as well as MeMan at high concentrations of >25 mm. At lower concentrations, an abrupt decline occurred to basically zero inhibitory power and this result was obtained repeatedly in all experiments performed.

To analyze the specificity of these effects, bacterial growth was tested after incubation with the employed glycosides at high concentrations (Table 2). In this assay we observed that all thiahexyl glycosides (**2**, **4**, and **6**) inhibit bacterial growth at high concentrations. Thus, the measured inhibitory effect could be shown to stem from toxicity rather than from carbohydrate-specific inhibition of adhesion. The question of why mannoside **2** shows the highest toxicity in this series cannot be answered at this stage, as *E. coli* bacteria have transporters for all three types of sugars, glucose, galactose, as well as mannose [55]. Recently, we have performed careful toxicity studies with various mannosides, which were designed as inhibitors of bacterial adhesion, and there little or no cytotoxicity was seen [56]. Interestingly, various biological effects have been reported for aminothiaalkyl-modified organic compounds, including antimicrobial activity [57,58,59,60].

Our study has important implications for further utilization of aminothiahexyl glycosides, regardless whether they are used as specific inhibitors or as control compounds in solution. As the aminothiahexyl aglycone is especially easily accessed in synthetic organic chemistry using the “thiol-ene” reaction of cysteamine to allyl glycosides, respective compounds are quite popular [61]. It has to be kept in mind that the aglycone part of a specific glycoside can not only alter its affinity to the bacterial lectin FimH but can lead to unexpected toxicity such in in the case of mannoside **2**. On the other hand, when glycosides such as **2**, **4**, or **6** are used to decorate surfaces, the resulting glycoarrays exert no cytotoxicity, suggesting that cellular uptake is a prerequisite for the effect (cf. Figure 3, Figure 4).

The effect of the assay set-up can be seen when data, which were obtained in the adhesion-inhibition assay are compared with those measured in the preincubation-inhibition-adhesion assay (Table 1). In the first case, a glycoside ligand has to compete for the carbohydrate binding sites of type 1-fimbriated *E. coli* with the highly mannosylated polysaccharide mannan. In contrast to this, in the preincubation assay the bacteria are initially exclusively presented to the glycoside ligand, and no competing compound is present in the first step of the assay. In accordance with these considerations, the IC_50_ of mannoside **1** is ~0.6 mm in the adhesion-inhibition assay, but 0.035 mm in the preincubation-inhibition-adhesion assay. An analogous observation was made with mannoside **2**. In addition, the IC_50_ of the glucoside **3** is ~120 mm in the first assay and ~50 in the second.

At this end it remains unclear, whether conformational rearrangement of FimH can result in altered carbohydrate specificity. In the herein performed tests, a “relaxed” conformation of the FimH carbohydrate-binding site must be assumed. As a next step, we will perform adhesion studies under tensile force, based on the herein reported results. After allosteric activation of FimH according to the catch bond mechanism, carbohydrate specificity of FimH will be tested accordingly.
